# Rhomboid proteases: key players at the cell surface within haloarchaea

**DOI:** 10.3389/fmicb.2025.1547649

**Published:** 2025-03-28

**Authors:** Mariana Inés Costa, Micaela Cerletti, Roberto Alejandro Paggi, Sofia Denise Frecha, Valeria Zoratti, Lucas Leonel Latorre, Rosana Esther De Castro, María Inés Giménez

**Affiliations:** Instituto de Investigaciones Biológicas, Universidad Nacional de Mar del Plata (UNMdP)-Consejo Nacional de Investigaciones Científicas y Técnicas (CONICET), Mar del Plata, Argentina

**Keywords:** *Haloferax volcanii*, rhomboid protease, haloarchaea, intramembrane proteases, archaeal morphology

## Abstract

**Introduction:**

Rhomboid proteases are intramembrane serine proteases that play a key role in regulating membrane proteins across all domains of life. However, their function in archaea remains poorly understood. The model halophilic archaeon Haloferax volcanii encodes two rhomboid homologs, rho1 (HVO_1474) and *rho2* (HVO_0727). Previous studies indicated that the deletion of rho2 resulted in mild alterations in motility, adhesion, biofilm formation, and cell morphology, suggesting potential functional compensation by *rho1*.

**Materials and methods:**

To investigate the role of these proteases, we generated single (Δ*rho1*) and double (Δ*rho1* Δ*rho2*) deletion mutants. Phenotypic characterization included viability assays, motility tests, adhesion and biofilm formation studies, as well as morphological analysis using microscopy. Functional overlap between rho1 and rho2 was evaluated through genetic complementation/overexpression experiments in which each gene was expressed in trans in the mutant backgrounds.

**Results:**

Both Δ*rho1* and Δ*rho1* Δrho2 mutants were viable, indicating that these genes are not essential in H. volcanii. The Δ*rho1* mutant exhibited increased motility, enhanced biofilm formation, reduced adhesion to glass surfaces, and significant morphological alterations, particularly in trace element-deficient conditions. The double mutant (Δ*rho1* Δ*rho2*) showed increased adhesion to surfaces, mild motility reduction, and fewer morphological abnormalities compared to Δ*rho1*. Complementation assays revealed that both rho1 and *rho2* could restore motility in Δ*rho2* and adhesion in Δrho1. However, only rho1 was able to complement the morphological defects, suggesting a degree of functional divergence between these homologs.

**Discussion:**

This work highlights the role of rhomboid proteases in regulating critical cell surface processes in *H. volcanii*, including biofilm formation, surface adhesion, and cell shape determination. The ability of rho1 and *rho2* to compensate for each other in certain functions while maintaining distinct roles underscores a complex regulatory interplay. Future research will focus on identifying natural substrates and elucidating the molecular mechanisms underlying rhomboid protease function in haloarchaea.

## Introduction

Over the past two decades there has been a growing interest in a distinctive group of proteases known as Intramembrane Proteases (IMPs), based on their remarkable ability to cleave peptide bonds within the hydrophobic environment of cellular membranes. These proteases are ubiquitously represented across all domains of life and serve diverse functions including protein maturation, activation of signaling molecules and protein degradation. Categorized according to their catalytic mechanisms, these proteolytic enzymes are classified into four families: serine, metallo, aspartyl, and a glutamyl protease (Kühnle et al., 2019).

The rhomboid protease (Rho) family includes intramembrane serine proteases and various pseudo proteases. Rho-like proteins are universally present throughout evolution, serving as regulators that influence the fate of membrane proteins (Kandel and Neal, 2020). Among Rho, those found in eukaryotes have been extensively studied for their crucial roles in regulating significant human pathologies such as male infertility (Radaelli et al., 2023), Parkinson’s disease (Shi et al., 2011), Alzheimer’s disease (Paschkowsky et al., 2016), type-2 diabetes (Walder et al., 2005; Civitarese et al., 2010) and cancer (Cheng et al., 2014; Song et al., 2015; Noy et al., 2016). Additionally, Rho from protozoan parasites causing amoebiasis, malaria, and toxoplasmosis play a role in promoting host cell invasion by cleaving surface proteins critical for the parasite entry into host cells (Brossier et al., 2005; O'Donnell et al., 2006; Baker et al., 2006; Baxt et al., 2008).

The biological role of Rho in prokaryotes physiology has been relatively underexplored. The *Escherichia coli* Rho homolog GlpG was the first to be investigated. GlpG can efficiently process eukaryotic Rho substrates showing protease activity (Reddy and Rainey, 2012). Furthermore, the crystal structure of the GlpG core domain has been resolved with a resolution of 2.1 Å (Wang et al., 2006). However, up to date, endogenous targets of GlpG have not been identified. The AarA Rho from the pathogenic bacterium *Providencia stuartii* specifically cleaves the N-terminal extension of TatA, a membrane-bound component of the twin-arginine protein translocation pathway. The processing of TatA activates the translocation mechanism, facilitating the export of an unidentified *quorum*-sensing signal (Stevenson et al., 2007). In Mycobacteria, null Rho mutants exhibit compromised biofilm formation and increased sensitivity to antibiotics (Kateete et al., 2012). *Bacillus subtilis* Rho YqgP cleaves the magnesium transporter MgtE. Additionally, YqgP interacts with the membrane-anchored metalloprotease FtsH which in turn completes the degradation of this polytopic membrane protein, resembling eukaryotic endoplasmic reticulum-associated protein degradation (Began et al., 2020). Rho of *Shigella sonnei* are involved in membrane protein quality control by specifically targeting “orphan” components of respiratory complexes. These components display protected metastable transmembrane domains when incorporated into a functional complex. Initial cleavage by Rho allows subsequent degradation of the orphan substrate (Liu et al., 2020). Quantitative shotgun mass spectrometry was used in *Corynebacterium glutamicum* to compare a Rho double mutant with the wild type (wt) strain. This study revealed differences in the abundance of proteins that participate in diverse cellular functions including a decrease in ribosomal subunits and RNA polymerase, variations in iron uptake proteins and changes in the abundance of lipid and mycolic acid biosynthetic enzymes, suggesting a functional link between Rho and cell envelope lipid biosynthesis. This connection was confirmed by shotgun lipidomics (Luenenschloss et al., 2022). Shotgun proteomics was also employed to compare a *Brucella abortus* Rho mutant with the parental strain. Proteins identified as potential substrates included denitrification enzymes and/or high oxygen affinity cytochrome c oxidase which are required for growth in low oxygen conditions (Marchesini et al., 2022).

Although Rho homologs are widely distributed in archaeal genomes, the biological relevance of this protease family in archaea remains poorly understood. The chromosome of the model haloarchaeon *H. volcanii* encodes two Rho homologs, designated *rho1* and *rho2*. A null mutation in *rho2* resulted in a strain (previously denoted as MIG1, and referred to as Δ*rho2* in this study) that exhibited reduced adhesion and motility, heightened sensitivity to novobiocin, impaired recovery from UV light irradiation and a truncation in glycosylation at N732 of the S-layer glycoprotein (Parente et al., 2014). Despite the significance and reproducibility of these phenotypes, they were relatively mild, suggesting potential functional complementation of the mutation by *rho1*. Comparative proteomics analysis between Δ*rho2* and the parental strain revealed that the absence of *Rho*2 had a widespread impact on the proteome, influencing the quantity and/or electrophoretic mobility of proteins associated with the observed phenotypes, as well as proteins involved in various processes such as metal homeostasis and cell division (Costa et al., 2018).

This study aimed to advance our understanding of the role of Rho in archaea. To achieve this, two novel deletion mutants were generated in *H. volcanii*: a single mutant targeting the *rho1* gene (Δ*rho1*) and a double mutant targeting both *rho1* and *rho2* (Δ*rho1* Δ*rho2*). Phenotypic characterization and functional cross-complementation assays revealed a partial functional overlap between the two proteases, along with a significant role for Rho in regulating cell surface physiology, impacting cell shape, motility, adhesion, and biofilm formation.

## Materials and methods

### Strains and growth conditions

Details regarding the strains, plasmids, and primers used in this study are shown in Supplementary Table S1. *H. volcanii* strains were cultured in CAB (de Silva et al., 2021), CA (CAB with no trace elements) or MGM media (Allers, 2009) at 42°C with agitation at 150 rpm. For the construction of growth curves, 3 mL starter cultures were inoculated from fresh isolated colonies and allowed to grow until the late exponential phase of growth (OD_600_ 0.7–1.2) was reached. Subsequently, 3 mL cultures were initiated using the starter cultures, at a final OD_600_ of 0.01 and allowed to grow under the aforementioned conditions. The OD_600_ was monitored at specified intervals using an Ultrospec10 cell density meter (Biochrom). The duplication time calculation and slope comparisons were performed using the GraphPad Prism version 8.0.2 for windows. For motility assays, *H. volcanii* strains were stab-inoculated in 0.25% (w/v) agar CAB plates and incubated at 42°C for 2–3 days. Motility was determined by measuring the diameter of the swimming ring with the ImageJ program.

### Generation of plasmid constructions

The chromosomal regions encompassing the ORFs of either the *rho1* (HVO_1474) or *rho2* (HVO_0727) genes were PCR-amplified using Fwrho1NdeI + Rvrho1BamHI (for *rho1*) and HVO0727NdeIF + HVO0727BamHIr (for *rho2*) primers (Supplementary Table S1) with iProof DNA polymerase (BioRad) and *H. volcanii* H26 genomic DNA as the template. The resulting amplicons (1,651 bp for *rho1* and 891 bp for *rho2*) were individually cloned into the TOPO-Blunt vector (Invitrogen). The identities of the cloned fragments were confirmed by sequencing (Macrogen, Korea). The fragments were then digested with NdeI and EcoRI (*rho1*) or NdeI and BamHI (*rho2*) (Thermo) and subsequently subcloned into the pTA963 vector (Allers et al., 2010), which had been digested with the same restriction enzymes. The ligation products were introduced into *E. coli* DH10β via transformation, passed through *E. coli* GM33 to obtain non-methylated plasmid DNA, and finally transformed into the indicated *H. volcanii* strains using the standard polyethylene glycol (PEG) transformation method (Allers, 2009).

### Construction of *H. volcanii rho1* in-frame knock-out mutants

The knock-out constructs were generated following a previously described protocol (Allers et al., 2004). Briefly, ~800 bp flanking each end of the *rho1* gene were PCR-amplified and consecutively cloned into the EcoRI/HindIII (upstream region) and the BamHI/XbaI (downstream region) sites of the haloarchaeal suicide vector pTA131. The resulting construct (pIG1) was initially amplified in *E. coli* DH10β and subsequently propagated in *E. coli* GM33 to obtain a non-methylated plasmid. This plasmid was then transformed into *H. volcanii* H26 or Δ*rho2* using the PEG method. Selection for a single homologous recombination event between one of the flanking regions on the knock-out construct and the chromosome (pop-in) was achieved by growth in CA medium lacking uracil (ura). Recombinants were subsequently cultured in liquid CA + ura (50 μg ml^−1^) with two passages to fresh medium to facilitate a second recombination event leading to the excision of the plasmid from the chromosome (pop-out). Liquid cultures were then plated on CA with ura (10 μg ml^−1^) and 5-FOA (50 μg ml^−1^), followed by incubation at 42°C for 5–10 days. Colonies were screened by PCR using the external primers (Fwverifycorto and Rvverify2020) to confirm elimination of *rho1*.

### Microscopy

Cells of *H. volcanii* H26 and mutant strains were inoculated from freshly isolated colonies in liquid medium until they reached the desired OD_600_. Culture aliquots (5 μL) were placed on a coverslip, and a 0.5% (w/v) agarose CAB plug (3 mm thick) was added on top. The cells were analyzed using phase-contrast microscopy at 100× magnification (Eclipse Ti-S, NIKON). Cells were segmented using Omnipose (Cutler et al., 2022) with a custom model partially trained on our images. Segmented cells were analyzed using CellProfiler 4.2.5 (Carpenter et al., 2006) and the area (μm^2^) and aspect ratio were determined. The aspect ratio, defined as the ratio of a cell’s major to minor axis, increases with cell elongation. Statistical analysis was performed using the Kruskal-Wallis test to assess differences among multiple groups. Subsequently, Dunn’s post-hoc test was employed to determine specific group differences. A *p*-value <0.001 was considered significant.

### Cell adhesion

Surface adhesion was assessed using the method described by Tripepi et al. (2010). *H. volcanii* cultures (3 mL) at an OD_600_ of ∼0.3 were incubated in 50 mL sterile screw-cap plastic tubes. Glass coverslips were inserted into each tube at a 90° angle and were incubated without shaking. After the specified time intervals coverslips were removed with forceps, submerged for 2 min in 2% (v/v) acetic acid and allowed to air dry. The dry coverslips were stained with 0.1% (w/v) crystal violet for 10 min, washed (three times) with distilled water, air-dried and then observed by light microscopy using a Nikon Eclipse Ti microscope. Cells were quantified using ImageJ.

### Biofilm formation

*H. volcanii* biofilms were formed on pre-sterilized 96-well flat-bottom polystyrene microtiter plates (6 wells for each strain) following the method described elsewhere (Shukla, 2017). In brief, 200 μL of liquid CAB cultures (OD_600_ ~ 0.3) were dispensed into each well and 200 μL of distilled water was added to peripheral wells to minimize water loss. The microtiter plate was then incubated for 72 h at 42°C. Planktonic cells were removed by inverting the plate onto absorbent paper and biofilms were fixed with 99% (v/v) methanol. The plates were washed twice with CAB and air-dried. Subsequently, 200 μL of a 0.2% (w/v) crystal violet solution was added to all wells. After 5 min excess crystal violet was removed and plates were washed with distilled water (4 times) before air drying. Finally, the cell-bound crystal violet was dissolved in 33% (v/v) acetic acid. Biofilm growth was monitored in terms of Absorbance at 570 nm (A_570_) using a microplate reader (BioTek ELx800).

## Results

### Rho homologs display functional complementation in *H. volcanii*

**Figure 1:**
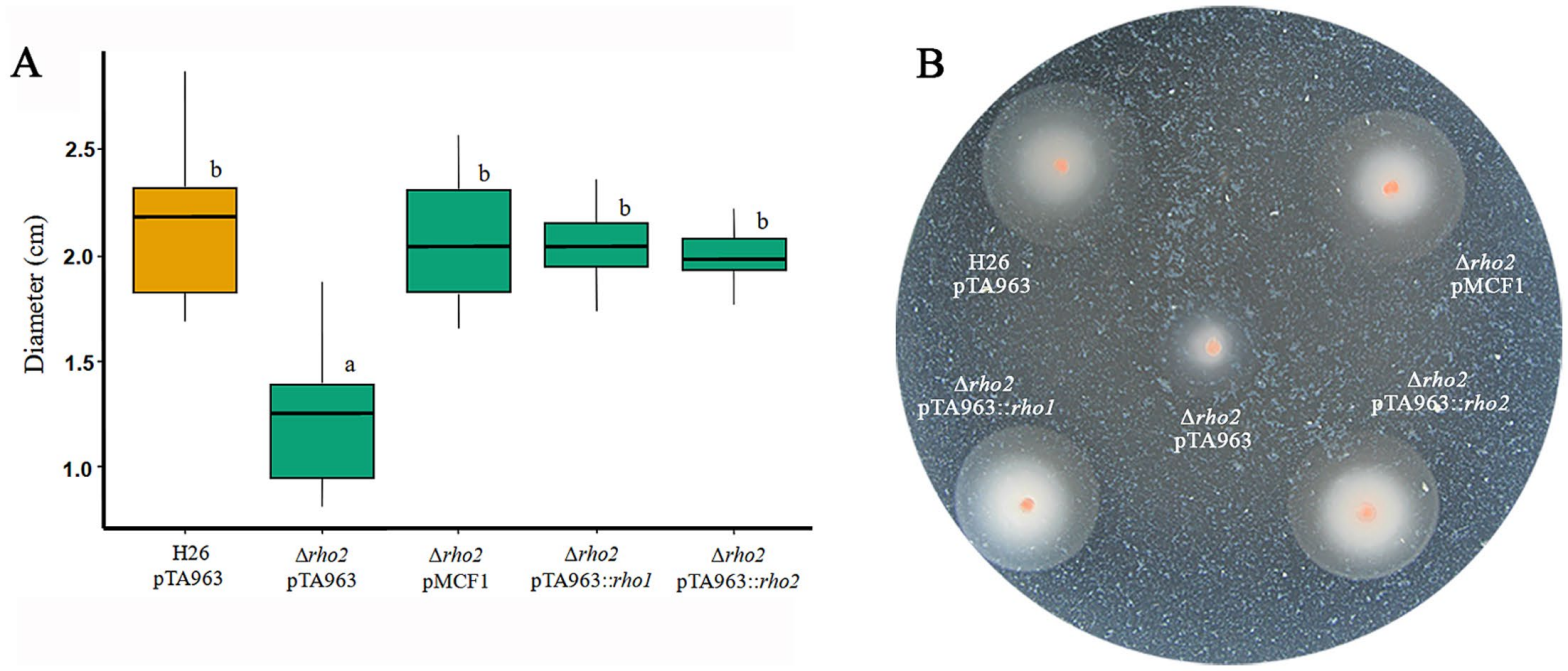
Complementation of the motility phenotype in the Δ*rho2* mutant with plasmids expressing *rho* homologs. *H. volcanii* H26 pTA963 and Δ*rho2* transformed with either pTA963, pTA963::*rho1*, pTA963::*rho2*, or pMCF1 (containing the complete *rho2*/*endV* operon, under the endogenous promoter) were cultured in CAB medium with 2 mM trp at 42°C overnight (ON) and subsequently adjusted to an OD_600_ = 0.5. For the motility assay, 1 μL of the adjusted culture was inoculated onto swimming agar plates with 2 mM trp and incubated for 48 h. Photographs were taken and the ring diameter was measured using ImageJ. All strains were inoculated on the same plate and three independent experiments with five replicates each were conducted. The results were analyzed using the R program. **(A)** Box plot of the distribution of the motility rings diameters for each strain. **(B)** Representative image of a soft agar motility plate.

The phenotypes exhibited by the Δ*rho2* deletion mutant were consistent but mild, suggesting a degree of functional complementation by the other *H. volcanii* Rho homolog, *rho1* (Parente et al., 2014). To test this, we focused on the most prominent Δ*rho2* phenotype: motility on soft agar plates. Motility assays were performed using the H26 and Δ*rho2* strains transformed with the empty pTA963 vector, as well as the Δ*rho2* strain transformed with pTA963 harboring either the *rho1* or *rho2* gene. The rhomboid gene is predicted to be part of an operon with the *endV*, which encodes for a type-V endonuclease. Additionally, a construct containing the pTA963 vector with the complete *rho2*/*endV* operon under the control of the endogenous promoter (pMCF) was included. This construct was previously reported to complement the motility defect of the Δ*rho2* mutant (Parente et al., 2014). As shown in Figure 1, the three constructs successfully restored motility to wt-type (H26) levels. However, in the case of the *rho1* construct, it is important to note that since an endogenous copy of this gene is present in the Δ*rho2* mutant, phenotype complementation is achieved through over expression by the addition of an extra copy of *rho1* from the pTA963 vector.

It is worth noting that the diameter of motility halos in a given strain is influenced by both growth (cell division) and motility (Palma et al., 2022). To distinguish between these factors, we compared the specific growth rates of the plasmid-transformed strains. The Δ*rho2* + pTA963 and Δ*rho2* + pTA963::*rho1* strains showed no significant variation in growth compared to the H26 + pTA963 control. In contrast, the Δ*rho2* + pTA963::*rho2* strain displayed a significant growth defect (*p* = 0.0007) (Supplementary Figure S1). Despite this, both Rho genes successfully complemented the swimming defect of the ∆*rho2* + pTA963 mutant. This indicates that, although the pTA963::*rho2* strain exhibited impaired growth, *rho2* expression from the plasmid was still sufficient to counteract growth reduction and fully restore motility to wt-type levels.

### Generation of single *rho1* and double *rho1 rho2* knock-out mutants

To identify the cellular processes regulated by Rho in *H. volcanii* we aimed to generate mutants devoid of the corresponding gene/s. Previously, we generated a single ∆*rho2* strain (Parente et al., 2014). In this work we constructed *H. volcanii* mutants lacking *rho1* in H26 (single *rho1* null mutant) or the Δ*rho2* strain (double null *rho1 rho2* mutant). Despite several initial unsuccessful attempts, we finally obtained the target strains by allowing one of the liquid medium passages (Materials and Methods section) to reach the stationary (S) growth phase (OD_600_ ~ 2). Gene deletion was confirmed via PCR using primers external to the knockout construct (Supplementary Figure S2) and the strains were denoted as “∆*rho1”* and *“∆rho1 ∆rho2.”* The successful generation of both mutant strains indicated that the presence of Rho is not required for *H. volcanii* viability at least under our standard laboratory conditions.

### Characterization of the mutant strains

#### Growth in liquid and solid media

**Figure 2:**
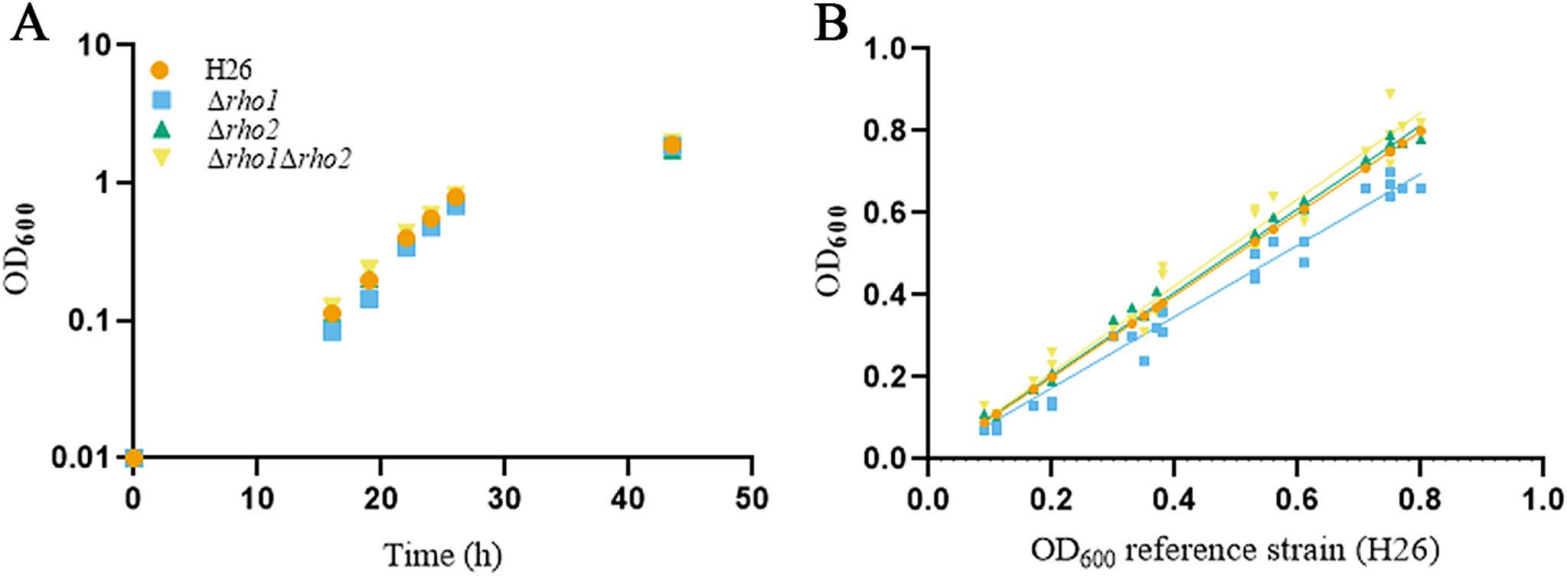
Evaluation of Rho deletion mutants growth in liquid medium. Cultures of the indicated strains were inoculated from ON starter cultures at an initial OD_600_ of 0.01 in 3 mL of CAB medium supplemented with ura (50 μg/mL). Growth was monitored by measuring OD_600_ at the indicated times and the values were used to generate the growth curve **(A)** Each culture was grown as three biological replicates, with experiments independently repeated at least three times. Growth rates were determined during the E phase by fitting linear regression models to the OD_600_ data and calculating the slopes, using GraphPad Prism software. For comparison, the H26, Δ*rho1,* Δ*rho2* and Δ*rho1* Δ*rho2* strains growth rate was plotted against the H26 growth curve, as a reference growth curve **(B)** Statistical significance was assessed using a two-tailed Student’s *t*-test (*p-*value <0.05 considered significant).

The growth of the mutant strains in liquid CAB medium was monitored by measuring OD_600_ over time (Figure 2A). While the growth curves of the ∆*rho2* and ∆*rho1* ∆*rho2* mutants did not exhibit differences compared to the parental H26 strain, the ∆*rho1* mutant showed a slight but statistically significant growth defect (*p*-value *=* 0.0276), with a duplication time of 4.27 h compared to 4.12 h for the H26 strain. This difference was evidenced by the variation in the slopes of the growth curves during the exponential (E) phase (Figure 2B). Additionally, growth was assessed under different salt conditions (4.8 M and 1.75 M NaCl) (Supplementary Figure S3). No significant differences were observed in the mutants’ response to suboptimal salt concentrations (1.75 M NaCl). At high salt concentrations (4.8 M NaCl), the Rho mutants showed a slight improvement in growth. However, when considering all experiments together (three independent assays, each performed in triplicate) the data exhibited substantial variability, and thus the results were deemed inconclusive. Colony size, morphology, and appearance on solid medium were indistinguishable from those of the H26 strain (data not shown).

#### Cell morphology

**Figure 3:**
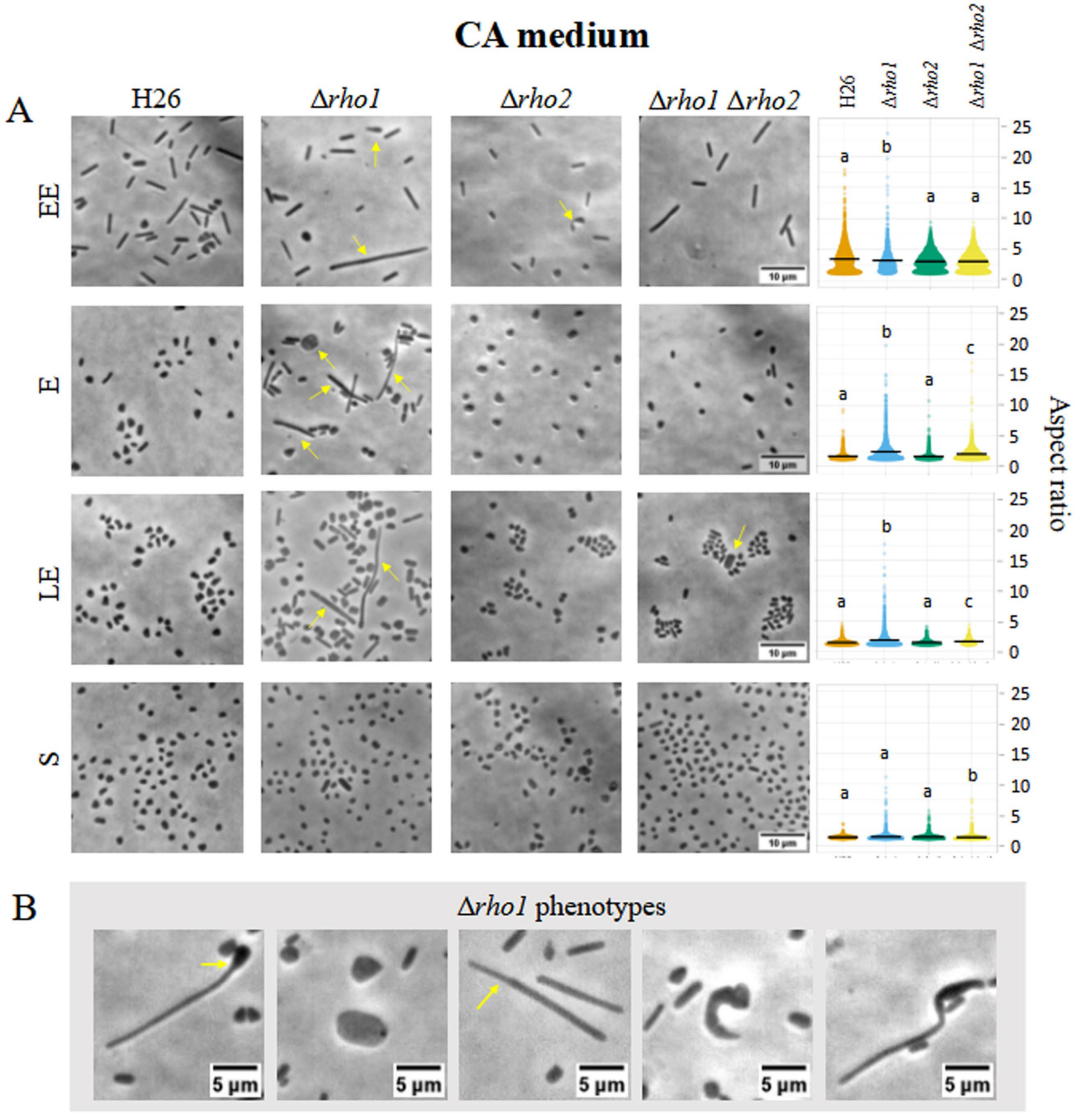
Cell morphology of *H. volcanii* Rho mutants in CA medium. *H. volcanii* single colonies of the specified strains were inoculated into 3 mL cultures of CA medium and samples were taken at EE (OD_600_ 0.1–0.3), E (OD_600_ 0.4–0.6), LE (OD_600_ 0.7–1), and S phases (OD_600_ ≥ 1.3). **(A)** Cells were imaged in an inverted microscope under 0.5% (w/v) CAB-agarose pads. Right panel shows the distribution of the cell’s aspect ratio for each strain and growth phase. At least 10 photograms from three independent cultures were analyzed for each strain and condition. **(B)** Different morphological abnormalities observed for the Δ*rho1* strain in CA medium.

**Figure 4:**
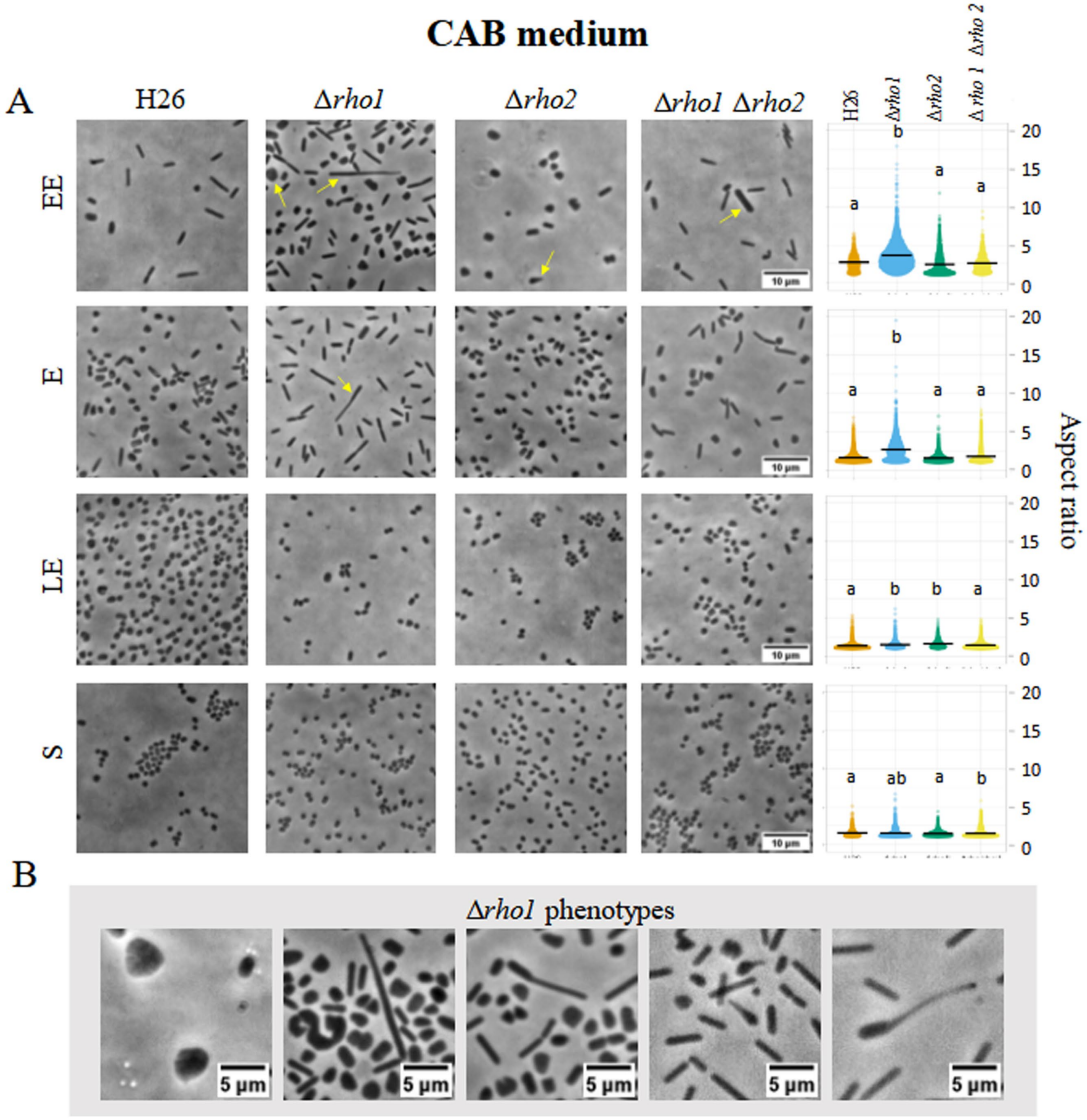
Cell morphology of *H. volcanii* Rho mutants in CAB medium. *H. volcanii* single colonies of the specified strains were inoculated into 3 mL cultures of CAB and samples were taken at the same stages of the growth curve depicted in Figure 3. **(A)** Cells were imaged in an inverted microscope under 0.5% (w/v) CAB-agarose pads. Right panel shows the distribution of cell aspect ratio for each strain and growth phase. At least 10 photograms from three independent cultures were analized for each strain and condition. **(B)** Morphological abnormalities observed for the Δ*rho1* strain in CAB medium.

To evaluate the impact of *rho* deletion on cell morphology, optical microscopy was used to compare *H. volcanii* H26 with Rho-deficient mutants (Δ*rho1*, Δ*rho2*, and Δ*rho1* Δ*rho2*) during early exponential (EE), E, late exponential (LE), and S growth phases in CA (Figure 3) and CAB media (Figure 4).

The Δ*rho1* strain exhibited pronounced morphological abnormalities, including elongated rods, giant cells, and drop-like or reed-like shapes (Figures 3B, 4B). These aberrations were particularly evident in the absence of trace elements, aligning with previous reports that cell shape in CA medium is affected by such deficiencies (de Silva et al., 2021). Consistent with this, some abnormal cells were also observed in the parental H26 strain when grown in CA. However, aberrant morphologies were markedly more frequent and severe in the Δ*rho1* mutant. Interestingly, these morphological defects resolved as cultures entered the S phase, where all strains reverted to the characteristic disk-shaped morphology (Figure 3A). In CAB medium, which is supplemented with trace elements, the Δ*rho1* mutant displayed aberrant morphologies primarily during the EE and E phases (Figure 4A). These abnormalities were less pronounced than those observed in CA and disappeared earlier in the growth curve, further underscoring the influence of trace element availability on cellular morphology.

The Δ*rho2* strain did not exhibit substantial morphological changes compared to the parental H26 strain, aside from occasional drop-like cells observed during the EE phase. Notably, the double mutant Δ*rho1* Δ*rho2* did not display the severe morphological defects seen in the Δ*rho1* single mutant under either growth condition (Figures 3A, 4A), suggesting the presence of compensatory mechanisms in the absence of both genes.

Cells were segmented and the aspect ratio determined using CellProfiler 4.2.5 (refer to Materials and Methods for detailed methodology). Consistent with the previous observations, the Δ*rho1* strain exhibited a higher average aspect ratio (lengthened cells) across all growth phases, except in the S phase where cells of all the strains predominantly looked like disks (Figures 3A, 4A, right panel). These differences were confirmed to be statistically significant according to Kruskal-Wallis and Dunn tests. In addition, the cell area of the different mutants was calculated and compared to that of the parental strain (Supplementary Figure S4). It was evident that the Δ*rho1* cells exhibited larger areas compared to the other strains throughout the E phase in CA medium. When cell areas were measured in the presence of trace elements (CAB), *rho1* elimination led to a milder increase in cell area with a similar effect observed in the double Δ*rho1* Δ*rho2* mutant during the EE and E stages of growth.

**Figure 5:**
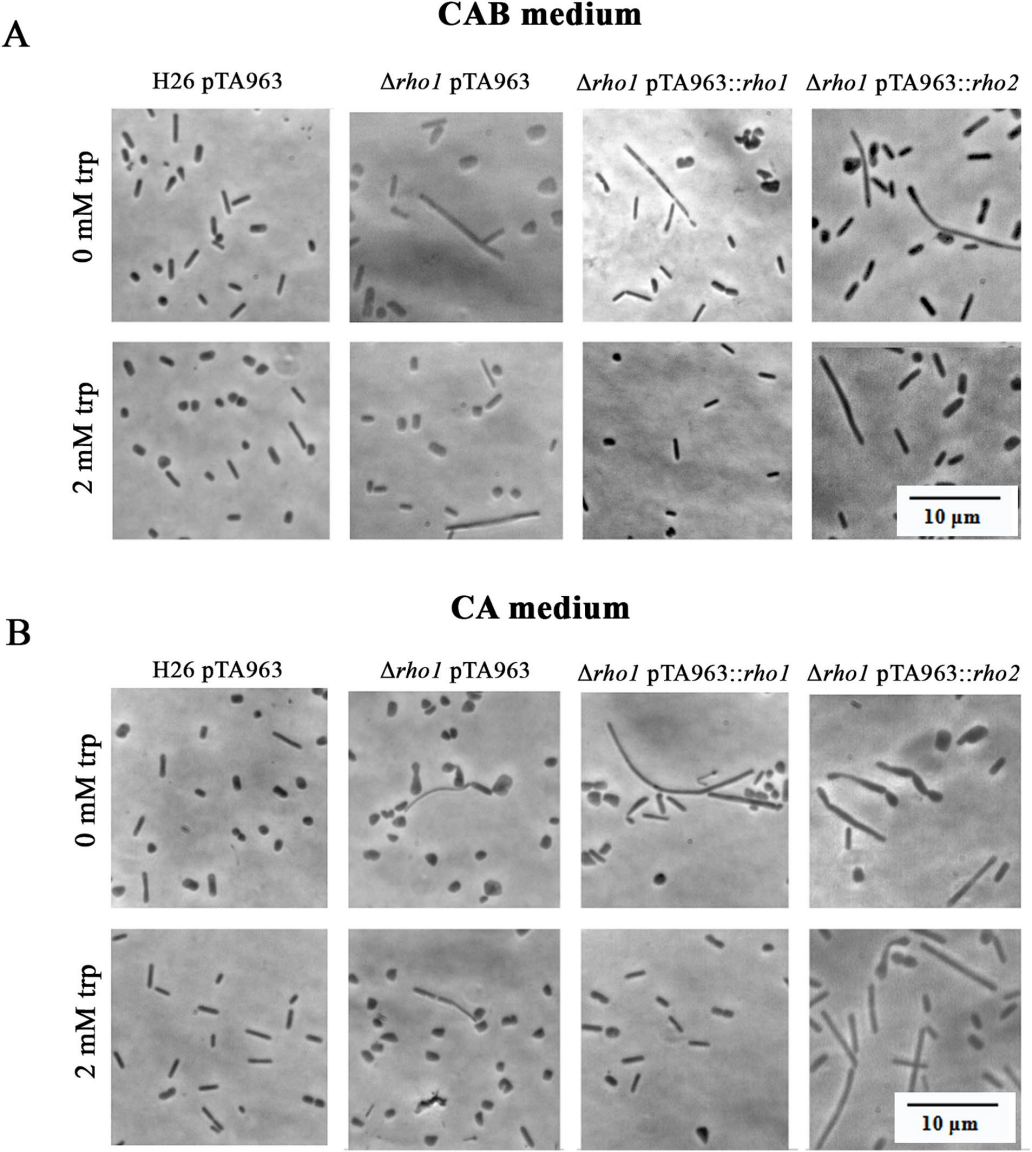
Complementation of the abnormal morphology phenotype in the Δ*rho1* strain. *H. volcanii* H26 and Δ*rho1* strains were transformed with either pTA963, pTA963::*rho1* or pTA963::*rho2* plasmids and cultured in CAB **(A)** or CA **(B)** medium until reaching the EE growth phase. Growth was conducted in the presence or absence of 2 mM trp to induce gene expression, as indicated on the left. Cell morphology was observed using optical microscopy as described in the Materials and Methods section. The photograms are representative of three independent cultures for each condition tested.

Morphological abnormalities in the Δ*rho1* strain were attributable to the loss of *rho1*, as expression of the gene from the pTA963::*rho1* vector under 2 mM tryptophan (trp) induction restored normal morphology in CAB and CA media (Figure 5). In contrast, *rho2* expression using the same conditions failed to rescue the phenotype, indicating that functional redundancy between these proteases is limited.

#### Motility on soft agar plates

**Figure 6:**
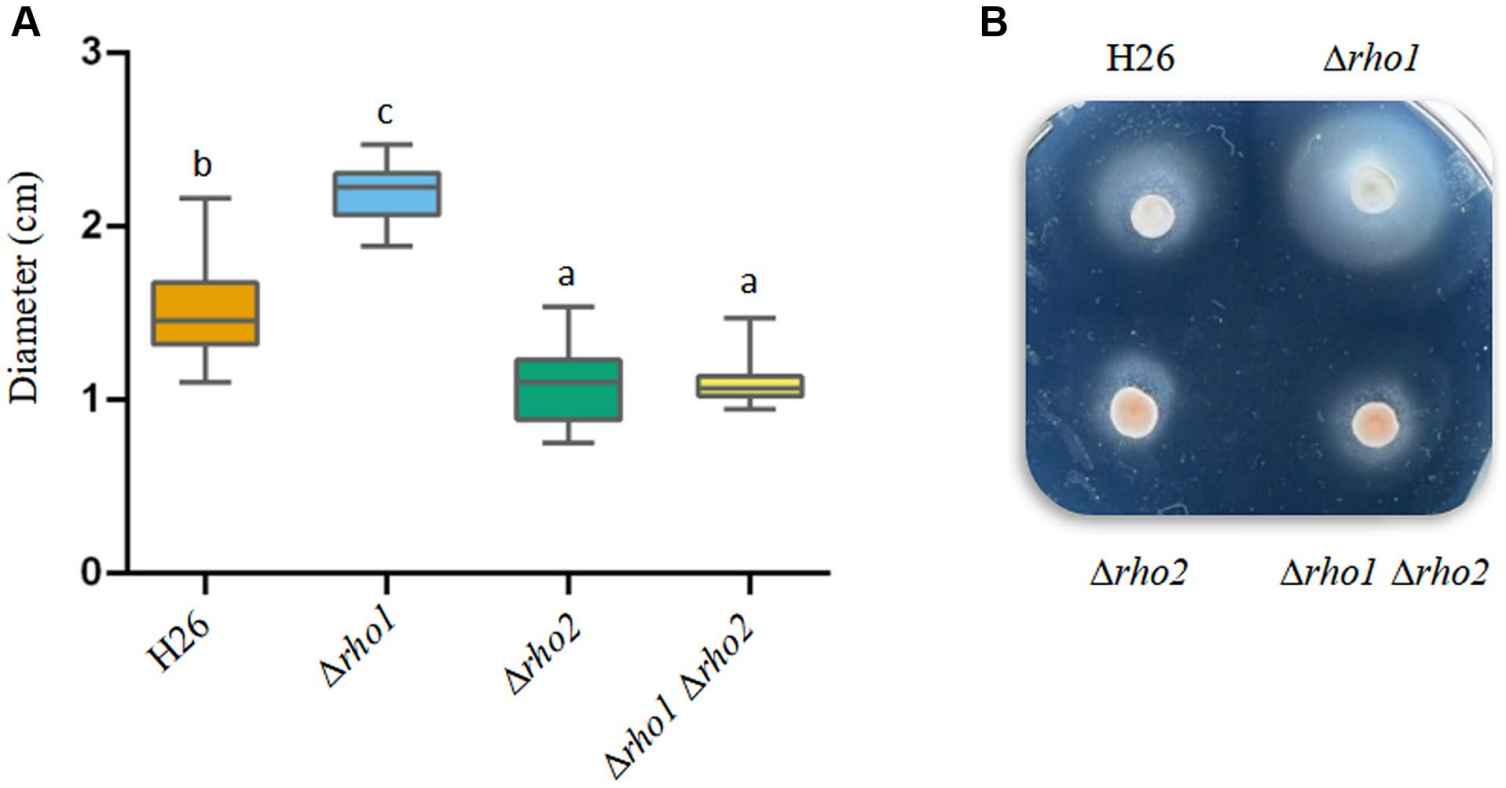
Effect of Rho gene elimination on cell motility in *H. volcanii*. Single colonies of the indicated strains were stab-inoculated on CAB soft agar plates containing 50 μg/mL ura and incubated at 42°C for 48–72 h. After incubation, photographs were taken, and the diameters of the growth halos were measured using ImageJ software. **(A)** Diameter values corresponding to three independent experiments, each with five replicates. Results were analyzed by LMMs (linear mixed models) with normal distribution to determine statistical significance between treatment and controls. A *post hoc* analysis was conducted using Tukey. All analyses were performed using R software version 3.6.1 (R Core Team, 2019). Statistically significant differences were determined at *p* < 0.05. **(B)** Representative photograph showing the motility halos generated by each strain.

As mentioned earlier the Δ*rho2* strain exhibited reduced motility, a defect that could be restored by overexpressing *rho1* (Figure 1). To assess whether Rho1 also influences swimming in *H. volcanii*, we evaluated the motility of the mutant strains generated in this study using soft agar plates. As shown in Figure 6, Δ*rho1* displayed larger swimming halos than the parental strain, suggesting enhanced motility compared to H26. Notably, given that the Δ*rho1* strain demonstrates a mild reduction in growth (Figure 2), the observation of larger swimming halos suggests that motility is even further enhanced in this mutant. Interestingly, the double mutant Δ*rho1* Δ*rho2* exhibited a slight decrease in motility similar to that of the single mutant Δ*rho2*. When these results were compared to those shown in Figure 1, it was noted that, although the Δ*rho2* mutant formed significantly reduced swimming halos in both assays, the reduction was more pronounced when comparing Δ*rho2* pTA963 with H26 pTA963 than when comparing the strains without the plasmid. This difference could be attributed to variations in media composition (CAB + ura for H26 and Δ*rho2* vs. CAB for plasmid-transformed strains) and/or a differential effect of the presence of the pTA963 vector between strains.

Complementation assays were attempted, however, the Δ*rho1* pTA963 strain did not show significant differences in the diameter of halos compared to the control strain (H26 pTA963), which contrasted with the differences observed between the same strains lacking the plasmid (data not shown). This discrepancy may be attributed to variations in growth rates which were more pronounced in strains harboring pTA963 (3 and 6% decrease in duplication time for Δ*rho1* and Δ*rho1* pTA963, respectively) and/or differential effects of the plasmid on the motility of H26 and Δ*rho1* strains. Consequently, functional complementation for this phenotype could not be investigated.

#### Cell adhesion and biofilm formation

**Figure 7:**
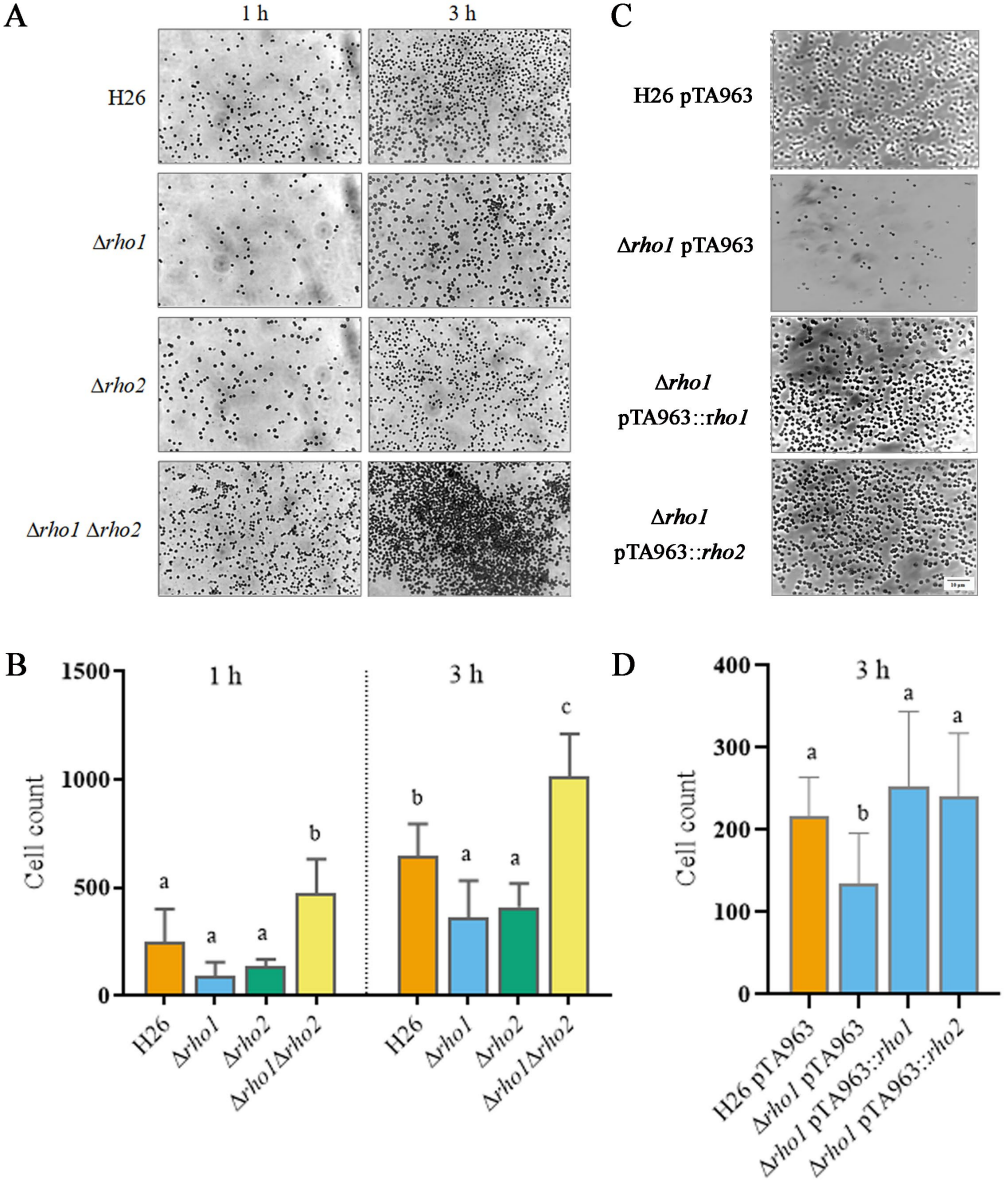
Evaluation of cell adhesion in Rho mutants. Glass coverslips were immersed in triplicate cultures of *H. volcanii* strains and incubated at 42°C for 1 or 3 h, as specified, followed by staining with crystal violet as described in the Materials and Methods section. **(A,C)** Optical microscopy images (1000×) showing cell adhesion to the glass surface for each strain. **(B,D)** Quantification of three independent experiments using Image J software. Data were analyzed using linear models (LM) with random effects, followed by Tukey’s post hoc test. Statistical analyses were performed with R software version 3.6.1 (R Core Team, 2019). Differences were considered statistically significant at *p* < 0.05.

We also assessed the capacity of the mutants to adhere to glass surfaces and to form biofilms. The Δ*rho1* strain evidenced a slightly reduced adhesion to glass coverslips similar to the Δ*rho2* strain. Surprisingly, the double mutant Δ*rho1* Δ*rho2* exhibited a significant increase in the number of adhered cells, suggesting a dysregulation of adhesion in the absence of both Rho homologs (Figures 7A,B). A copy of the *rho1* gene expressed from pTA963 vector could restore the H26 phenotype confirming that the reduced number of adhered cells was due to the absence of this gene (Figures 7C,D). Complementation of this phenotype was also accomplished by introducing an extra copy of the other Rho gene homolog (*rho2*) in the Trp-inducible expression vector pTA963, indicating functional overlap between these proteases (Figures 7C,D).

All the strains showed the ability to form immersed liquid biofilms (Supplementary Figure S5A) and exhibited honeycomb patterns when removing the Petri dish lid (Supplementary Figure S5B), as previously described for other *H. volcanii* strains (Schiller et al., 2020).

**Figure 8:**
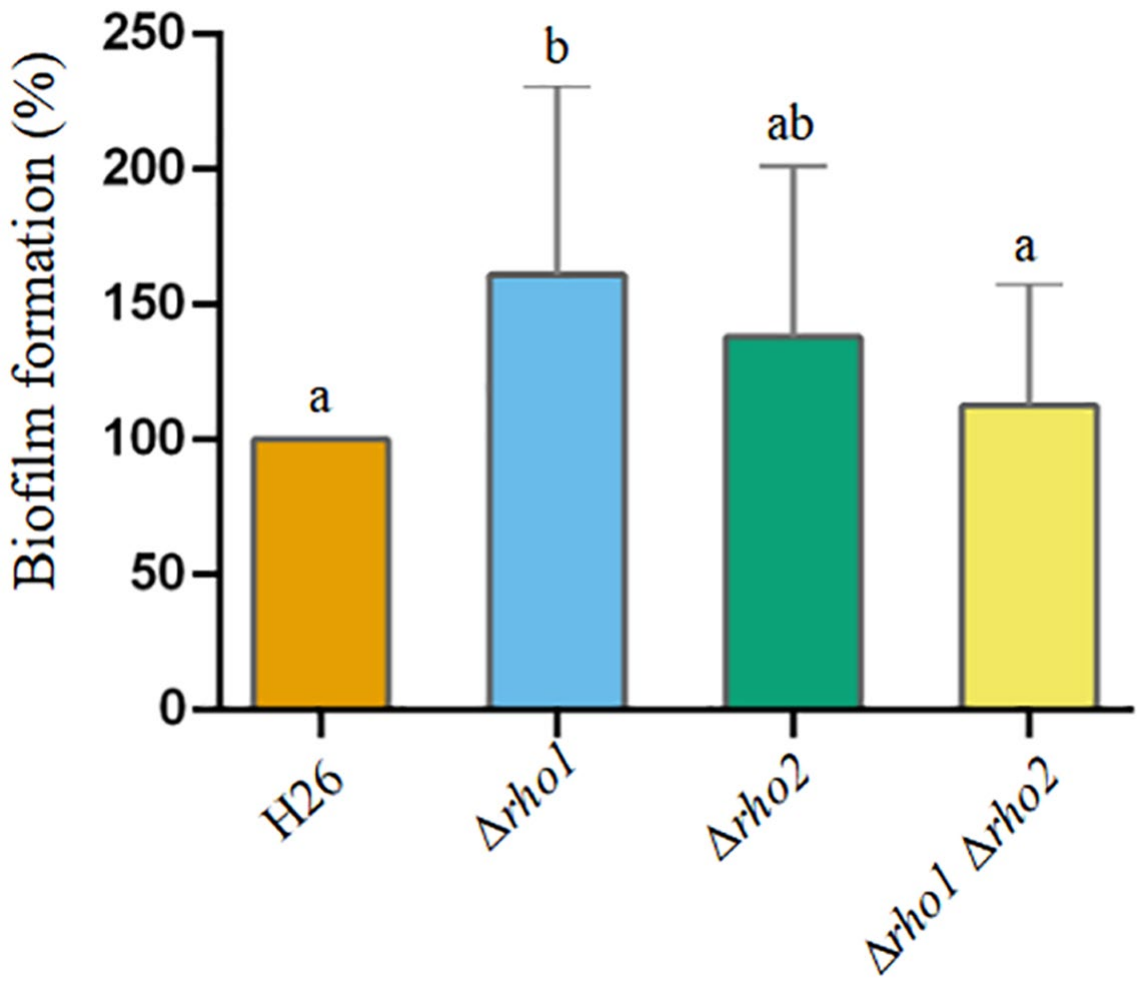
Effect of Rho gene deletion on biofilm formation in *H. volcanii*. EE phase cultures were transferred to 96-well polystyrene plates and incubated without agitation at 42°C for 72 h. Early-stage biofilms were stained with crystal violet according to the Materials and Methods section. Data was obtained from three independent experiments, each including six technical replicates. The amount of cell-bound colorant was quantified by measuring A_574_, and values corresponding to the mutant strains were normalized to the average value of the parental strain (H26). Results were analyzed by LMMs (linear mixed models) with normal distribution to determine statistical significance between treatment and controls. A post hoc analysis was conducted using Tukey. All analyses were performed using R software version 3.6.1 (R Core Team, 2019). Statistically significant differences were determined at *p* < 0.05.

Early biofilm formation on plastic surfaces was assessed using the crystal violet method (Figure 8). The results showed that the single mutant Δ*rho1* exhibited enhanced capacity for biofilm formation compared to the parental strain and the other two mutants (Δ*rho2* and Δ*rho1* Δ*rho2*). Attempts to complement this phenotype by introducing the *rho1* gene in trans were unsuccessful, as the Δ*rho1* mutant carrying the pTA963 plasmid displayed a significant reduction in biofilm formation relative to the H26 pTA963 control (not shown). These findings, combined with the results from motility assays (see above), suggest that the presence of the pTA963 vector differentially influences the behavior of the H26 and Δ*rho1* strains.

## Discussion

Rho are regulatory IMPs highly represented throughout archaeal, bacterial and eukaryotic genomes, however, the understanding of their biological role in archaea still remains limited. In this study, we used complementation assays, gene knockout, and mutant phenotypic characterization to gain insight into the relevance of the Rho family in haloarchaea.

The archaeal structure responsible for cell motility in *H. volcanii* is the archaellum. It consists of two proteins: Arch1, the primary archaellum component, and Arch2, which is less abundant and plays a regulatory role (Tripepi et al., 2013). Deletion of *arch1* renders *H. volcanii* cells non-motile (Tripepi et al., 2012). Interestingly, proteomic studies conducted by our group showed downregulation of *arch1 in the ∆rho2* mutant compared to the wt strain (Costa et al., 2018), consistent with the reduced motility phenotype observed previously (Parente et al., 2014). In addition, the protein levels of PibD, a peptidase involved in archaellins and pilins maturation and several glycosylases (involved in archaellin/pilin glycosylation) are also affected in the Δ*rho2* strain (Costa et al., 2018). In this study, we confirmed that the overexpression of a copy of *rho1* from a plasmid restores motility in the Δ*rho2* strain to the same extent as the *rho2* gene or a construct containing the complete operon *rho2/endV* (Figure 1), indicating functional complementation between Rho homologs. This observation suggests that despite differences in sequence, topology and functional domain organization (Parente et al., 2014), Rho1 and Rho2 probably participate in the regulation of common pathways. Consequently, to gain further insights into the function of Rho in haloarchaea, we generated two additional deletion mutants: a knockout in *rho1* (∆*rho1*) and a double mutant affecting both *rho1* and *rho2* (∆*rho1* ∆*rho2*).

The successful generation of both mutants implied that the genes encoding Rho are not essential for viability in *H. volcanii* under our standard laboratory conditions. It is noteworthy that the knockouts were only attainable when at least one of the three passages in liquid medium intended to alleviate the selection pressure (pop-out) reached at the late S growth phase. Our observations revealed altered cell morphology in the ∆*rho1* mutants during the E phase (Figures 3, 4) and these atypical cells may be outcompeted by those reverting to the wt genotype. Cells lacking *rho1* revert to a normal morphology during the S phase and therefore, mutant cells at this stage may not have a fitness disadvantage compared to the wt. This could facilitate the detection of mutant colonies on selective medium plates when at least one of the successive cultures reached the S phase. This hypothesis is supported by studies conducted in *M. smegmatis* Rho mutants, which showed normal growth in liquid medium but were outcompeted by the wt strain when they were co-cultured (Kateete et al., 2012). It is also possible that the mutants obtained in this work carried not only the desired mutation but also secondary mutations that might act as second site suppressors. However, restoration of wt phenotypes (Figures 5, 7C,D) upon complementation with *rho1* in trans suggests that the phenotypes were specifically caused by the absence of *rho1*.

Archaeal cells exhibit a diverse array of distinctive morphologies including cocci (e.g., Borrell et al., 2023), rods (e.g., Strakova et al., 2023), squares (Walsby, 1980) and triangles (Takashina et al., 1990). *H. volcanii* displays motile rod-shaped cells in the EE/E growth phases which transition to flat, non-motile disks upon reaching LE and S phases (de Silva et al., 2021). These morphological changes likely represent an adaptation to variations in environmental conditions at each growth stage. However, the mechanisms underlying these changes in cell shape remain incompletely understood.

In this work, we showed that the Δ*rho1* mutant displays a spectrum of aberrant cell shapes during the E phase including giant cells, elongated filaments, irregular morphologies, drop-like forms as well as reed-like (tubular) shapes (Figures 3B, 4B). Some of these morphologies resemble those observed in mutants lacking tubulin-like homologs FtsZ1 and FtsZ2 as well as the divisome-interacting protein SepF (Lliao et al., 2021; Nussbaum et al., 2021) suggesting compromised cell division in the Δ*rho1* strain. Recently, two halofilins (HalA and HalB) proposed to contribute to the disk-to-rod transition in *H. volcanii* were described (Curtis et al., 2024). It is noteworthy that perturbations in cell shape observed in the Δ*rho1* strain evidenced some similarity to those of the *halB* null mutant. However, since the biological targets of Rho1 have yet to be identified, it is still not possible to determine at which point of *H. volcanii* cell morphology regulation this protease is involved and thus, explain the phenotypic similarities to the aforementioned mutants. Ongoing work will help to clarify this issue.

The irregular cells of the Δ*rho1* strain persisted during the E phase but reverted as cultures progressed to the LE (CAB medium with trace elements) or S phase (CA medium without trace elements) (see Figures 3A, 4A). These observations were consistent with our hypothesis aiming at the occurrence of a defect in cell division which may be overcome at the S phase when the cells are not actively duplicating. On the other hand, the differences observed between CA and CAB media are not surprising as the positive influence of trace elements on cell shape stability in *H. volcanii* has been previously documented by de Silva et al. (2021). Complementation of the abnormal cell shape phenotype by *rho1* expression from pTA963 vector was successful confirming that the variations in cell shape are a consequence of gene deletion. Cross complementation with an extra copy of the *rho2* homolog was unsuccessful, indicating that Rho2 is not involved in the generation of the cell shape phenotype (Figure 5).

We also investigated the effects of *rho2* elimination and the double gene knockout (Δ*rho1* Δ*rho2*) on cell morphology in CA and CAB media (Figures 3A, 4A). The Δ*rho2* mutant did not exhibit significant differences in morphology compared to the parental strain consistent with our previous findings in a nutrient-rich medium (Parente et al., 2014). Intriguingly, the double mutant showed a mild impact on cell morphology primarily reflected in slight differences in aspect ratio and area measurements compared to the parental strain, with significantly fewer occurrences of cells with abnormal phenotypes compared to the Δ*rho1* strain (Figures 3A, 4A). These observations suggest the existence of compensatory mechanisms regulating cell morphology when both Rho homologs are absent in *H. volcanii*. In addition to the findings presented in this study, previous research has reported the involvement of Rho in cell division and morphology determination in bacteria. Studies on *P. stuartii* and *B. subtilis* evidenced that Rho deletion mutants showed atypical filamentous cell shapes (Rather and Orosz, 1994; Mesak et al., 2004) indicating the relevance of Rho in prokaryotic cell shape determination.

The impact of Rho gene deletion on motility, adhesion, and early biofilm formation was also evaluated in this study. The Δ*rho1* mutant exhibited increased motility on soft agar plates (Figure 6), suggesting that Rho1 and Rho2 regulate motility in opposing ways, as Rho2 deficiency reduces swimming motility (Parente et al., 2014; Figure 1). This finding contrasts with the ability of *rho1* overexpression to complement the Δ*rho2* motility defect (Figure 1). If the absence of *rho1* enhances motility (Figure 6), one would expect its overexpression to reduce swimming halos. The results obtained in this work suggest that both proteins are likely part of a regulatory network controlling motility. Since the double mutant exhibits a motility defect comparable to that of the ∆*rho2* mutant (Figure 6), it can be speculated that Rho1 functions downstream of Rho2 within this network. Additionally, the ability of either *rho1* or *rho2* to restore motility in both mutants suggests a degree of functional redundancy or a compensatory mechanism, which becomes evident when rhomboid genes are overexpressed. Future studies aimed at identifying potential substrates and/or interacting partners of Rho1 and Rho2 will help clarify this regulatory interplay.

*H. volcanii* can grow in biofilms which exhibit some differential traits, this includes changes in cellular morphology and an unusual form of social motility (Chimileski et al., 2014). The initial step of biofilm development involves adhesion to the abiotic surface and microcolony formation which depends on the presence and glycosylation of type-4 pilins (Esquivel et al., 2013, 2016). In this study, we investigated the role of Rho in cell adhesion and early-stage biofilm formation. We found that both single mutants Δ*rho1* and Δ*rho2* showed a mild effect on cell adhesion, evidenced by a reduced number of cells bound to glass during a 1–3 h incubation period (Figure 6; Costa et al., 2018). In previous work, we showed that Δ*rho2* was affected in pilin content (Costa et al., 2018) and had a defect in protein glycosylation (Parente et al., 2014), observations that possibly explain the reduced adhesion to glass surfaces. Surprisingly, the double mutant showed a significant increase in the number of cells adhering to the glass coverslip (Figure 7). These results suggest that both proteases regulate the first step of biofilm biogenesis; it is likely that one Rho homolog is sufficient to modulate the level/processing of a regulatory factor that promotes cell adhesion and consequently, when both proteases are absent, this factor is dysregulated resulting in augmented number of bound cells. This is in agreement with the observation that both Rho genes are able to complement this phenotype when an extra copy is expressed from a plasmid (Figures 7C,D).

When the later stages of biofilm formation were assessed (72 h), this effect was no longer observed. The double mutant showed biofilm levels comparable to the parental strain, while the single Δ*rho1* mutant exhibited a slight increase in biofilm formation (Figure 8). Although these results may appear contradictory to those shown in Figure 7, it is important to note that these assays evaluate different stages of biofilm development. The adhesion assay focuses on the initial attachment phase, occurring within the first few hours of incubation with the coverslip, whereas the biofilm assay measures overall biofilm formation, including microcolony development and maturation, after 72 h of incubation. The transition from attachment to maturation is likely governed by distinct regulatory mechanisms, in which Rho proteins may play a role at different stages or in the transition itself. Further investigation will be needed to clarify the specific pathways involved. The involvement of Rho proteins in biofilm formation has been previously reported in *Mycobacterium smegmatis*. This Gram-positive bacterium encodes two Rho homologs, and in this organism, single deletion mutants exhibited reduced biofilm formation, while the double mutant displayed biofilm levels comparable to the wt-type strain (Kateete et al., 2012).

It is important to note that complementation (and cross-complementation with *rho2*) of the *rho1* motility and biofilm generation phenotypes could not be achieved. When the corresponding assays were attempted, strains harboring the empty pTA963 vector (used as experimental controls) did not exhibit the same behavior as the non-transformed strains. No differences were observed in motility on soft agar plates or in the formation of 72 h biofilms between H26 pTA963 and Δ*rho1* pTA963, in contrast to the variations observed between H26 and Δ*rho1* (Figures 6, 8). This may be explained by the presence of pTA963, as it has been reported that simply carrying a plasmid may affect cell metabolism, size, shape and growth of a given strain (Abdul-Halim et al., 2015; Patro et al., 2023). In this work, we observed that the Δ*rho1* pTA963 strain exhibited a more severe growth defect compared to H26 pTA963, in contrast to the smaller growth differences between Δ*rho1* and H26. These variations in growth may impact motility and/or biofilm formation. Additionally, the presence of pTA963 may also directly influence these processes. Consistent with this possibility, the Δ*rho2* mutant displayed a greater reduction in motility compared to control strains when transformed with the pTA963 vector (Figures 1, 6). Since this mutant does not exhibit growth differences relative to the wt strain (regardless of whether it carries the pTA963 vector, Figure 2), it is likely that the observed effect results from the plasmid differentially impacting the mutant and parental strain or from the presence/absence of ura (used as the plasmid selection marker) in the growth medium. These findings warrant further investigation.

Altogether, the data presented in this work provide evidence of the relevance of Rho in key processes occurring at the haloarchaeal cell surface including motility, cell adhesion, biofilm generation and cell shape determination. In addition, cross-complementation (homolog overexpression) assays indicated that even though Rho1 and Rho2 are involved in the regulation of related processes and to some extent display functional overlap, they also have distinctive roles, as for example regulation of cell shape. Ongoing research aims to identify and confirm natural targets of *H. volcanii* Rho1 and Rho2, which will contribute to clarify the specific role of these proteases in (halo)archaeal physiology and cell surface proteostasis.

## Data Availability

The original results are included in the article/Supplementary material, further inquiries can be directed to the corresponding author.
