# Supplementary figures and images for: Rhomboid proteases: key players at the cell surface within haloarchaea
Source: Front Microbiol. 2025 Mar 28;16:1547649. doi: 10.3389/fmicb.2025.1547649 (PMC11985538; doi:10.3389/fmicb.2025.1547649)

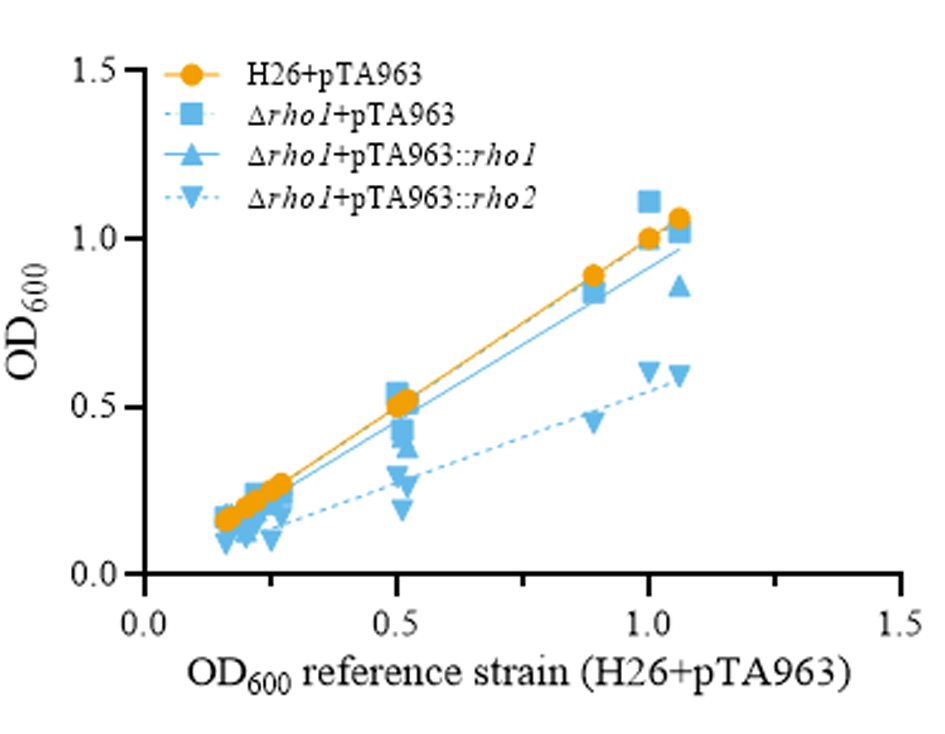

Supplement: Supplementary file 1 [file Image_1.tif]

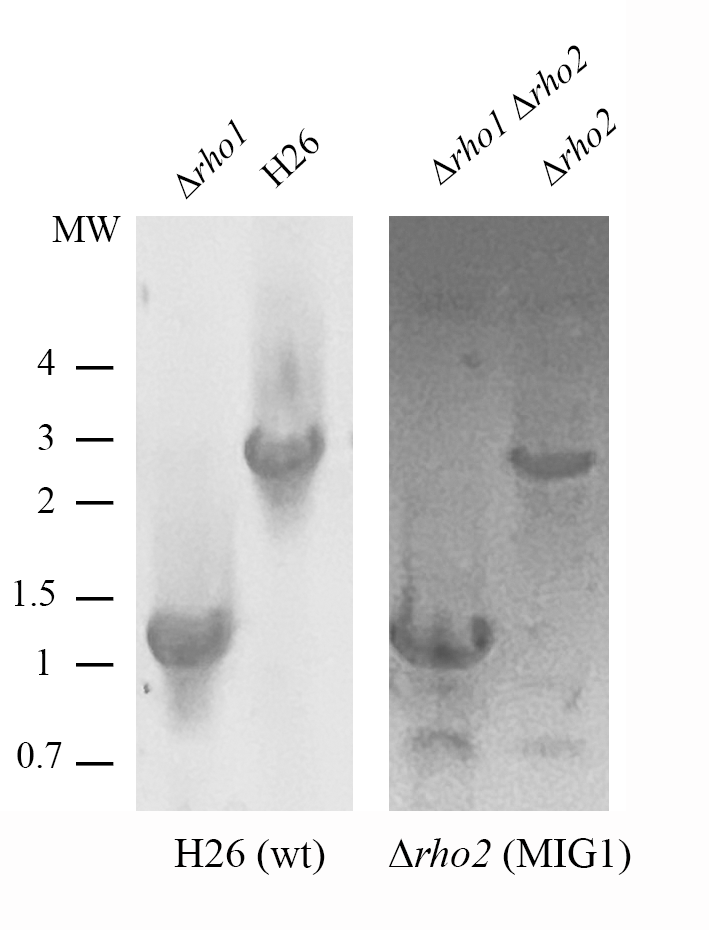

Supplement: Supplementary file 2 [file Image_2.tif]

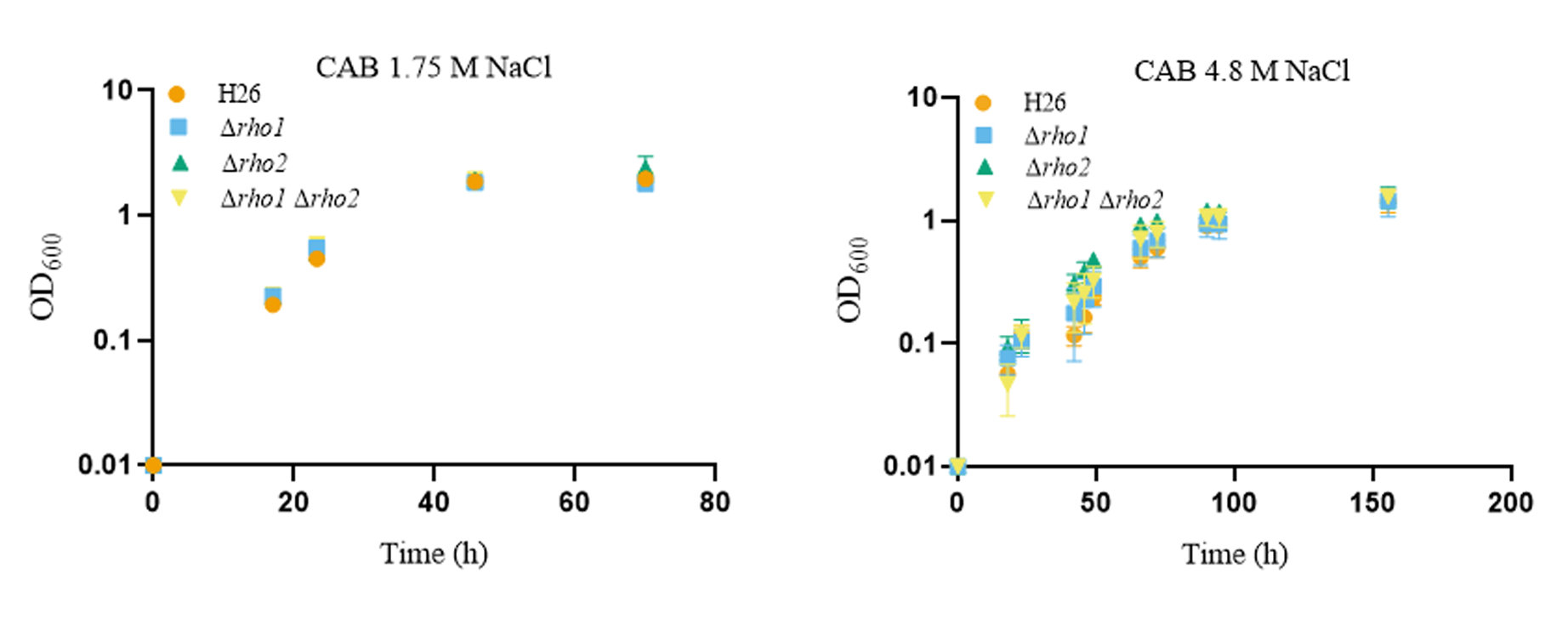

Supplement: Supplementary file 3 [file Image_3.tif]

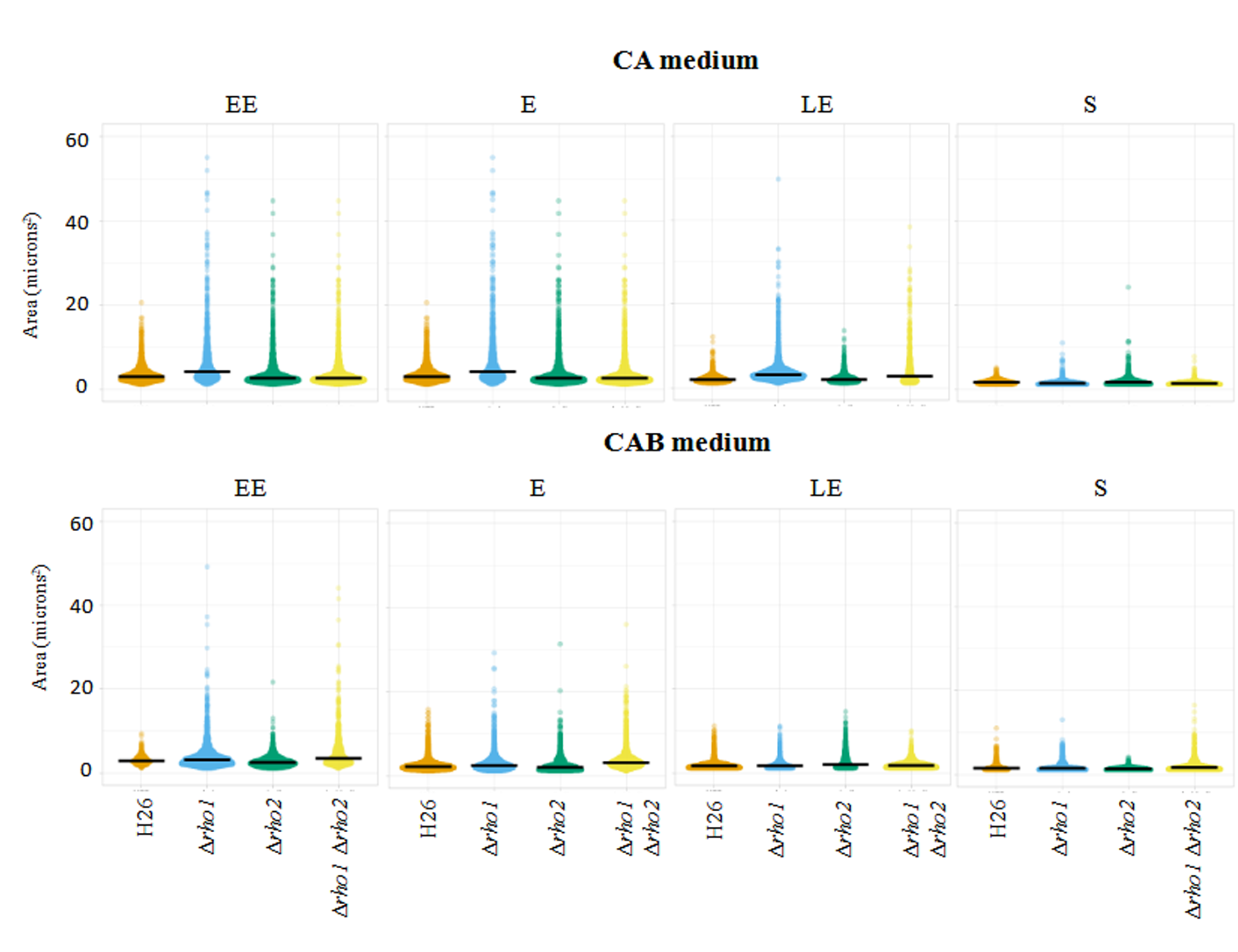

Supplement: Supplementary file 4 [file Image_4.tif]

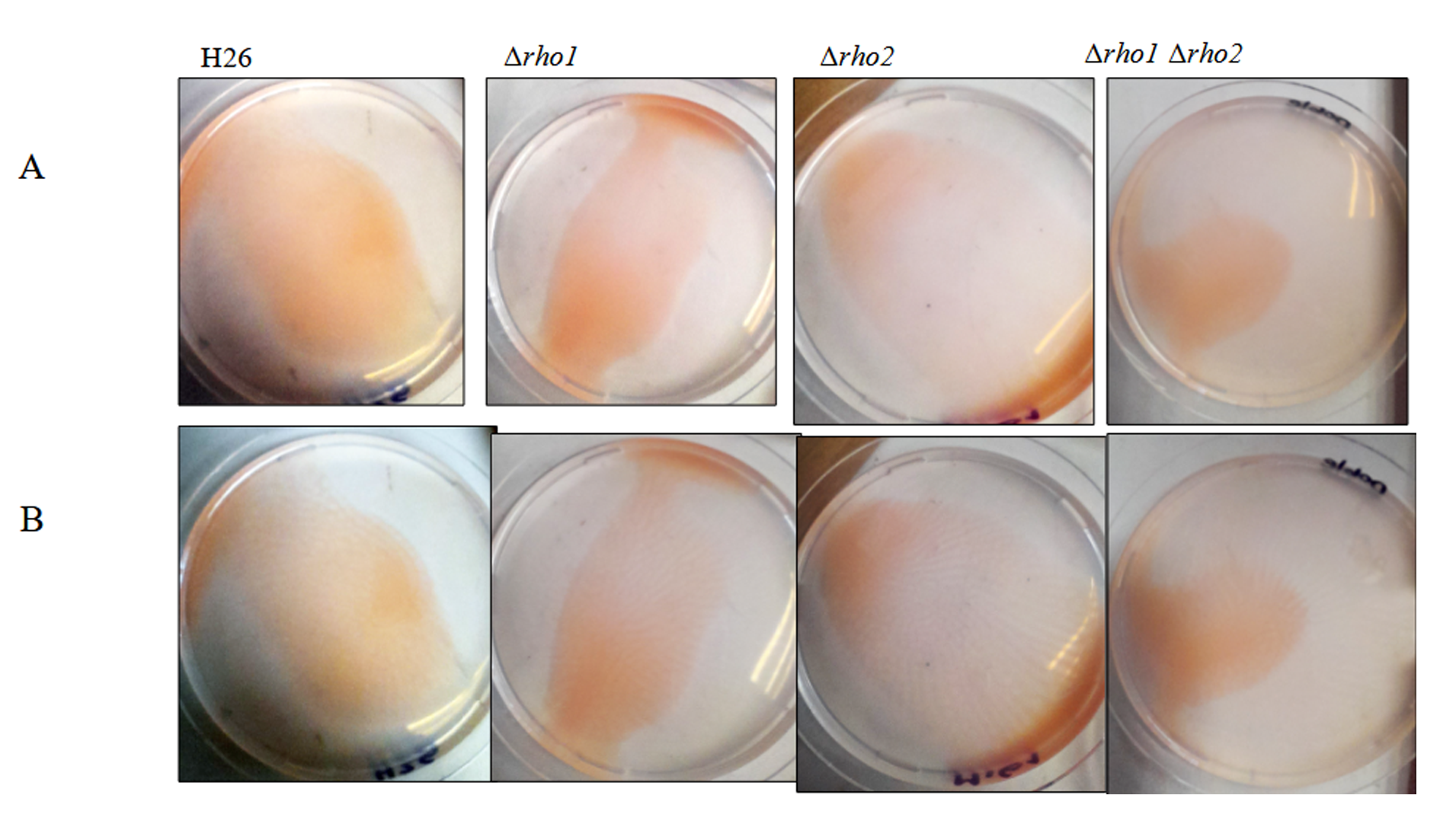

Supplement: Supplementary file 5 [file Image_5.tif]
