# Supplementary material for: Rhomboid proteases: key players at the cell surface within haloarchaea
Source: Front Microbiol. 2025 Mar 28;16:1547649. doi: 10.3389/fmicb.2025.1547649 (PMC11985538; doi:10.3389/fmicb.2025.1547649)
Supplement: Supplementary file 6 [file Table_1.docx]

| **Strains** | | |
| --- | --- | --- |
| Name | Description | Reference |
| *Haloferax volcanii* | | |
| H26 | *Haloferax volcanii* DS70 pyrE2 (-) | Allers *et al.,* 2004 |
| Δ*rho2* (MIG1) | H26 *rho2* (-) | Parente *et al*., 2014 |
| Δ*rho1* | H26 *rho1* (-) | This work |
| Δ*rho1* Δ*rho2* | H26 *rho1* (-) *rho2* (-) | This work |
| *Escherichia coli* | | |
| DH10β | F- mcrA Δ(mrr-hsdRMS-mcrBC) Φ80lacZΔM15 ∆lacX74 endA1 recA1 deo^R^Δ (ara leu)7697 araD139 galU galA nupG rpsLλ- | Grant *et al*., 1990 |
| GM33 | LAM-, IN(rrnD-rrnE)1, F^−^ dam-3 sup-85 (Am) | Marinus, 1973 |
| **Plasmids** | | |
| TopoBlunt |  | Invitrogen |
| pTA963 | Amp^R^, overexpression vector with His_6_ tag, *pyrE2* and *hdrB* markers, and pHV2 origin. | Allers *et al*., 2010 |
| pTA963::*rho1* | pTA963 with *rho1* (HVO_1474) inserted in the NdeI/BamHI restriction sites | This work |
| PTA963::*rho2* | pTA963 with *rho2* (HVO_0727) inserted in the NdeI/BamHI restriction sites | This work |
| pMCF1 | pTA963 with region 207 bp upstream of Hvo_0727 start codon and 103 bp downstream of Hvo_0726 stop codon, cloned between the ApaI and BamHI sites. | Parente *et al*., 2014 |
| pTA131 | Amp^r^; pBluescript II containing *Pfdx-pyrE2* | Allers *et al.,* 2004 |
| pIG1 | pTA131 with 799 bp upstream of *rho1* inserted in the HindIII/EcoRI restriction sites and 815 bp downstream of *rhoI* inserted in the BamHI/XbaI restriction sites | This work |
| **Primers*** | | |
| Fw*rho1*NdeI | ‘5 - gCATATGctagagataccgggctg - 3’ | This work |
| Rv*rho1*BamHI | ‘5 - attaGGATCCctaattgtacgtctcctgtc - 3’ | This work |
| FwUPrho1HindIII | ‘5 - tgAAGCTTactaaccgaacgtaa - 3’ | This work |
| RvUPrho1EcoRI | ´5- gGAATTCgagctacctgtgtcgt - 3´ | This work |
| FwDwrho1BamHI | ´5- atGGATCCggcgcggtcggcgggc - 3´ | This work |
| RvDwrho1XbaI | ´5- taTCTAGAacgtcgcggagtgctgt -3´ | This work |
| Fwverifycorto | ´5- caagcgtatggaggagttg -3´ | This work |
| Rvverify2020 | ´5- acctccgcgacctgaa -3´ | This work |
| HVO0727NdeIF | 5´-agCATATGatgcctatctgcgacgt-3´ | This work |
| HVO0727BamHIr | 5´-gtGGATCCtcagaaccgaccgcggccg-3´ | This work |

* Restriction sites are indicated in uppercase

**References**

ALLERS, T., BARAK, S., LIDDELL, S., WARDELL, K. & MEVARECH, M. 2010. Improved strains and plasmid vectors for conditional overexpression of His-tagged proteins in *Haloferax volcanii.* Appl Environ Microbiol, 76, 1759-69.

ALLERS, T., NGO, H. P., MEVARECH, M. & LLOYD, R. G. 2004. Development of additional selectable markers for the halophilic archaeon *Haloferax volcanii* based on the leuB and trpA genes. Appl Environ Microbiol, 70, 943-53.

Grant, S. G. [Jessee](https://pubmed.ncbi.nlm.nih.gov/?sort=pubdate&term=Jessee+J&cauthor_id=2162051), J., [Bloom](https://pubmed.ncbi.nlm.nih.gov/?sort=pubdate&term=Bloom+FR&cauthor_id=2162051), [F. R.](https://pubmed.ncbi.nlm.nih.gov/?sort=pubdate&term=Bloom+FR&cauthor_id=2162051) & [Hanahan](https://pubmed.ncbi.nlm.nih.gov/?sort=pubdate&term=Hanahan+D&cauthor_id=2162051) [D.](https://pubmed.ncbi.nlm.nih.gov/?sort=pubdate&term=Hanahan+D&cauthor_id=2162051) 1990. Differential plasmid rescue from transgenic mouse DNAs into *Escherichia coli* methylation-restriction mutants. Proc. Natl. Acad. Sci. U.S.A. 87 4645-9.

Marinus, M. G. 1973. Location of DNA methylation genes on the *Escherichia coli* K-12 genetic map. Mol. Gen. Genet. 127, 47–55.

PARENTE, J., CASABUONO, A., FERRARI, M. C., PAGGI, R. A., DE CASTRO, R. E., COUTO, A. S. & GIMENEZ, M. I. 2014. A rhomboid protease gene deletion affects a novel oligosaccharide N-linked to the S-layer glycoprotein of *Haloferax volcanii*. J Biol Chem, 289, 11304-11317.
