# Supplementary material for: Rhomboid proteases: key players at the cell surface within haloarchaea
Source: Front Microbiol. 2025 Mar 28;16:1547649. doi: 10.3389/fmicb.2025.1547649 (PMC11985538; doi:10.3389/fmicb.2025.1547649)
Supplement: Supplementary file 7 [file Table_2.docx]

**Supplemental Figures legends**

**Figure S1. Comparison of growth rates between *H. volcanii* strains harbouring plasmid constructs.** Cultures of the indicated strains were inoculated from overnight (ON) starter cultures at an initial OD_600_ of 0.01 in 3 mL of CAB medium supplemented with 2 mM trp. Growth was monitored by measuring OD_600_ at different times over a 31 h period. Each culture was grown in three biological replicates, with experiments independently repeated at least three times. Growth rates were determined during the exponential phase by fitting linear regression models to the OD_600_ data and calculating the slopes, using GraphPad Prism software. For comparison, the strains growth rate was plotted against the H26 pTA963 as a reference growth curve. Statistical significance was assessed using a two-tailed Student’s *t*-test (*P* < 0.05 considered significant).

**Figure S2. PCR verification of *rho1* deletion from the chromosome of *H. volcanii* H26 and Δ*rho2* (MIG1).** Colonies resulting from the pop-out of the pTA131 vector were analyzed using PCR with primers external to the knockout construct (Fwverifycorto and Rvverify2020, Table S1), followed by electrophoresis on an agarose 0.7% (w/v) gel. The parental strain (H26 or Δ*rho2*) is indicated at the bottom. In each case, a representative colony that reverted to the parental genotype (expected amplicon size 2,826 bp) or that eliminated the *rho1* gene (expected amplicon size 1,176 bp) is shown. The migration of molecular weight markers (Inbio Highway) is indicated in kb on the left (MW).

**Figure S3. Growth of *H. volcanii* rhomboid mutants in liquid media with different NaCl concentrations.** *H. volcanii* single colonies of the specified strains were inoculated into 3 ml starter cultures of CAB medium with uracil (50 μg/mL). Once the cultures reached late log phase, they were used to inoculate triplicate 3 ml cultures in CAB containing the indicated NaCl concentration at an OD_600_ of 0.01 and allowed to grow at 42°C and 150 rpm. At the specified time points, the OD_600_ of each culture was monitored and used to construct the growth curves. The results shown are representative of at least three independent experiments.

**Figure S4. Area distribution of rhomboid mutant cells through *H. volcanii* growth.** Single colonies of the specified strains were inoculated into 3 ml cultures of CA (A) or CAB (B) medium and samples were taken at the same stages of the growth curve depicted in Figs. 3 and 4, observed by phase contrast microscopy (1000x) and photographed. Cell area was determined as described in the Materials and Methods section.

**Figure S5. Immersed liquid biofilm formation**. *H. volcanii* cultures of the indicated strains were grown in liquid cultures up to stationary phase (OD_600_ ̴ 2), poured in plastic sterile Petri dishes and incubated without agitation at 42 ֩C for 18 h. **A**. Representative images of immersed liquid biofilms formed by the different strains. **B.** Honeycomb pattern formed after removing the lid of the Petri dish for 1-2 min. The images are representative of 3 independent experiments.
